# Risk Assessment of Opioid Misuse in Italian Patients with Chronic Noncancer Pain

**DOI:** 10.1155/2014/584986

**Published:** 2014-08-10

**Authors:** Renata Ferrari, Genni Duse, Michela Capraro, Marco Visentin

**Affiliations:** ^1^Division of Psychology, San Bortolo Hospital, Via Rodolfi, 36100 Vicenza, Italy; ^2^Pain Management Unit, S. Antonio Hospital, Padua, Italy; ^3^Pain Management and Palliative Care Unit, San Bortolo Hospital, Vicenza, Italy

## Abstract

*Objective*. Opioid therapy in patients with chronic noncancer pain must be preceded by evaluation of the risk of opioid misuse. The aim of this study was to evaluate the predictive validity of the Italian translation of the Pain Medication Questionnaire (PMQ) and of the Diagnosis Intractability Risk and Efficacy Score (DIRE) in chronic pain patients. *Design*. 75 chronic noncancer pain patients treated with opioids were enrolled and followed longitudinally. Risk of opioid misuse was evaluated through PMQ, DIRE, and the physician's clinical evaluation. Pain experience and psychological characteristics were assessed through specific self-report instruments. At follow-ups, pain intensity, aberrant drug behaviors, and presence of the prescribed opioid and of illegal substances in urine were also checked. *Results*. PMQ demonstrated good internal consistency (Cronbach's *α* = 0.77) and test-retest reliability (*r* = 0.86). Significant correlations were found between higher PMQ scores and the number of aberrant drug behaviors detected at 2-, 4-, and 6-month follow-ups (*P* < 0.01). Also the DIRE demonstrated good predictive validity. *Conclusions*. The results obtained with specific tools are more reliable than the clinician's evaluation alone in predicting the risk of opioid misuse; regular monitoring and psychological intervention will contribute to improving compliance and outcome of long-term opioid use.

## 1. Introduction

The problem of poorly controlled pain is still considerable: in Europe 19% of adults suffer from continuous pain that seriously compromises the quality of their emotional, social, and working life [1]. Opioids represent an important option for pain management; while there is agreement on their use in acute and cancer pain, long-term use for noncancer chronic pain remains controversial [2]. Indeed, some patients benefit from such treatment in terms of pain reduction and improvement in quality of life, while others do not [3]. Side effects, absence of any improvement in physical function, misuse, abuse, and addiction are relatively common during chronic opioid administration. The American literature reports overall rates of opioid misuse and abuse ranging from 4% to 26% [4, 5]. Such different rates may be due to a lack of a universally accepted definition of terms that describe the various types of incorrect behavior consequent to chronic opioid prescription. It is obvious that a clear and accepted terminology is necessary for improving communication and statistics.

In this paper we use the terms* misuse* and* abuse* according to the following definitions [6].* Substance misuse* is the use of any drug in a manner other than how it is indicated or prescribed, while* substance abuse* is defined as the use of any substance when such use is unlawful or when it is detrimental to the user or to others.

Moreover, the prevalence may depend also on the different populations tested. In Italy there are no data about opioid misuse and abuse since, until recently, opioids were not prescribed but in a small percentage of terminal patients [1, 7], due to regulatory and cultural barriers. In clinical practice we often find that patients tend to avoid the use of opioids even if prescribed by specialists; as a consequence, the health authorities have expressed very little concern about this problem until now.

Risk factors for opioid abuse and addiction can be divided into three categories: genetic, substance-related, and psychosocial factors. Patients with a personal or family history of substance abuse and with one or more psychosocial issues (such as anxiety, depression, personality disorders, or childhood abuse) are at greater risk of developing addiction, especially if the treatment is not carefully structured [3].

The aim of pain treatment is effective pain relief and the consequent functional improvement. Therefore, specialists are concerned about the possibility of abuse as well as about the lack of compliance with the analgesic treatment, including the patient's decision to decrease the prescribed dosage or to stop the treatment. For this reason, the detection of aberrant drug-related behaviors seems to be mandatory. An aberrant drug-related behavior is defined [8] as any medication-related behaviors that depart from strict adherence to the prescribed therapeutic plan of care. In line with this definition, an aberrant behavior is not only an abuse behavior, but also any behaviors not in accordance with the therapeutic plan or that may hinder it.

Guidelines recommend that the use of opioids in patients with chronic noncancer pain must be preceded by an assessment of potential benefits and risks of aberrant drug-related behaviors and should include a psychological and psychiatric assessment [2, 9].

In recent years, many tools have been developed for this purpose [10, 11]: among them, the Pain Medication Questionnaire (PMQ) [12] and the Diagnosis Intractability Risk and Efficacy (DIRE) Score [13] were created specifically for chronic pain patients. The PMQ is a self-report screening instrument that can easily be integrated into clinical-care routine. In the original version reliability coefficients were acceptable, and higher PMQ scores were found to be correlated with a high risk of opioid misuse. In a further study, significant differences in the mean PMQ score were found in patients with a history of substance abuse compared with patients without such a history; moreover, higher PMQ scores were found in patients who interrupted opioid treatment [14]. The DIRE is an assessment tool used in a multidisciplinary setting that requires a medical and psychological assessment of the patient. The psychometric analysis of the original version of the DIRE showed good internal consistency and significant correlations between DIRE score and measures of compliance and efficacy of long-term opioid therapy.

The present study examines the psychometric properties and clinical utility of the Italian translation of the PMQ and the DIRE. The predicting validity of the two tools was assessed by (a) the numbers of aberrant drug behaviors at 2-, 4-, and 6-month follow-ups and (b) the positivity of urine toxicology screening performed at 2- and 6-month follow-ups.

The scores obtained with the two instruments were also compared with the clinical evaluation made by the physicians in the pretreatment phase based on their subjective impression.

Furthermore, as psychosocial distress has been previously found to be a relevant risk factor in the development of opioid misuse [3, 12], the relationship between high/low-PMQ and DIRE scores and measures of anxiety, depression, and personality traits was also investigated.

## 2. Materials and Methods

### 2.1. Subjects

88 consecutive patients meeting the inclusion criteria were referred to the Pain Management Units of San Bortolo Hospital in Vicenza and Sant'Antonio Hospital in Padua, between December 2009 and January 2012. Inclusion criteria were age between 18 and 70, presence of noncancer pain for at least 6 months, a score of 4 or over in the last month average pain intensity as assessed on an 11-point Numerical Rating Scale, good knowledge/understanding of Italian, absence of cognitive deficit, pain not controlled by weak opioids or other pharmacological and nonpharmacological treatments used for at least 3 months, and informed consent to participate in the study. Of these, 75 agreed to participate in the study (11 refused to participate in the study, while 2 patients were excluded for not filling out the questionnaires properly); the sociodemographic and clinical characteristics of the group of subjects are represented in Table 1.

### 2.2. Instruments

The* Pain Medication Questionnaire* (PMQ) [12] is a self-administered questionnaire that describes a series of dysfunctional behaviors and characteristics in patients using analgesics. The tool consists of 26 items, for each of which the subject must indicate their degree of agreement on a 5-point Likert scale. A total score is obtained by adding the scores of the single items. High scores are correlated with a high risk of opioid misuse. In particular, scores of 25 or over are predictive of opioid misuse, while scores of 30 or over suggest that the patient should be frequently monitored during treatment [15].

The* Diagnosis Intractability Risk and Efficacy *(DIRE)* Score *[13] is a tool that consists of 4 factors: diagnosis, intractability, risk, and efficacy. Each factor requires assessment on a 3-point scale, where a score of 1 corresponds to characteristics and behaviors indicative of a negative prognosis and a score of 3 is indicative of suitability for treatment with opioids. The risk factor comprises four subcategories: psychological health, chemical health, reliability, and social support. The total score can vary from a minimum of 7 to a maximum of 21; higher scores (≥14) are predictive of good patient compliance and treatment efficacy.

The* medical risk evaluation *requires the physician to provide a clinical estimate based on subjective impression, of the risk of opioid misuse answering 4 questions on an 11-point numerical scale: (a) compliance with medical treatment (0 = no compliance; 10 = maximum compliance), (b) risk of abuse, (c) risk of underuse of the drug (0 = no risk, 10 = maximum risk), and (d) expected efficacy of treatment (0 = no efficacy, 10 = maximum efficacy).

The* aberrant drug behaviors* (ADB): based on the current literature [8], a list of 20 aberrant drug behaviors—that indicate a broad array of problematic nonadherence behaviors—was created. The physician must tick those behaviors displayed by the patient at 2-4- and 6-month follow-ups; examples of such behaviors are “the patient uses other opioids in addition to those prescribed” and “the patient displays little interest in managing himself and his rehabilitation.”

*Urine toxicology screening *was performed with fast immunodosages that could be read visually, allowing the qualitative determination of the pharmacological substances and their metabolites present in the urine. For opiates, marijuana, and buprenorphine, QuikStrip OneStep immunodosages by Syntron Bioresearch were used; QuikPac IITM OneStep was used to detect the presence of amphetamines, methamphetamines, cocaine, and oxycodone. In case of uncertainty the urine sample was sent to the laboratory for gas chromatography confirmation.

The psychological assessment included a structured interview and the following tools.

The* Visual Analogue Scale *(VAS) consists of a 10 cm long horizontal line, the start and end points of which are labeled “no pain” and “worst possible pain,” respectively. The patient is asked to mark the points corresponding to his/her worst, least, and average pain intensity in the last month.

The* Minnesota Multiphasic Personality Inventory II *(MMPI-2) [16, 17] is a 567-true-false-item self-report questionnaire which assesses psychiatric symptoms and personality organization; after checking for validity indicators (in order to screen out invalid protocols) only the ten basic clinical scales were used to indicate different psychological conditions.

The* Beck Depression Inventory II *(BDI-II) [18, 19] is a 21-item self-administered questionnaire used to determine the patient's depressive reaction, assessing both the cognitive and the somatic components.

The* State Trait Anxiety Inventory-Y *(STAI-Y) [20, 21] is a 40-item questionnaire that assesses the level of patient anxiety; two scores can be obtained referring to state and trait anxiety.

Finally, the* Pain Related Self-Statement Scale *(PRSS) [22, 23] is a self-administered scale developed to assess the cognitions specifically triggered in pain situations that might inhibit or promote coping responses. Two total scores can be obtained for the subscales catastrophizing and coping.

### 2.3. Procedure

This is an observational, prospective, and longitudinal study, and it consisted of the following assessment phases: patient selection, collection of pretreatment data, and 2-, 4-, and 6-month follow-ups. The specialist physician selected patients according to the clinical criteria indicated above. Pretreatment data were collected in the two-week period following selection. It included the filling-out of the PMQ by the patient and of the DIRE by the multidisciplinary team. Questionnaires to assess the pain intensity (VAS), coping strategies (PRSS), affective/emotional condition (STAI-Y, BDI-II), and personality characteristics (MMPI-2) were also administered in pretreatment assessment. Medical and psychological follow-ups were scheduled 2, 4, and 6 months after the start of treatment and included the filling-out of ADB and the readministration of VAS, STAI, and BDI-II. At each follow-up session patients were also required to collect a urine specimen which was immediately examined by the same person in both centers; in case of uncertainty the urine sample was sent to the laboratory for gas chromatography confirmation.

Both PMQ and DIRE were translated by the authors, according to the guidelines for the process of cross-cultural adaptation of self-report measures, and approved by the first author of each instrument.

The study protocol was approved by the Institutional Ethic Committee of both hospitals.

### 2.4. Statistical Analysis

Continuous variables are expressed with mean, standard deviation, minimum and maximum, and centiles, when appropriate. As the studied variables were not normally distributed (assessed by Kolmogorov-Smirnov test), to examine the differences between continuous variables, Kruskal-Wallis and Kolmogorov-Smirnov nonparametric analyses of variance tests were used; Chi-square test was used for the comparison of frequency distributions. Repeated measures ANOVA was used to analyze the presence of significant differences in pain intensity among patients at high/low risk of opioid misuse. Bonferroni correction was used in post hoc analysis to control for type II errors. The reliability was assessed through test-retest Pearson's *r* (PMQ) and internal consistency was determined by Cronbach's *α* (PMQ and DIRE). Spearman's nonparametric coefficient was used to examine the relationship between the PMQ and DIRE scores and the number of aberrant drug behaviours detected at the medical follow-up.

## 3. Results and Discussion

### 3.1. Results

**Figure 1:**
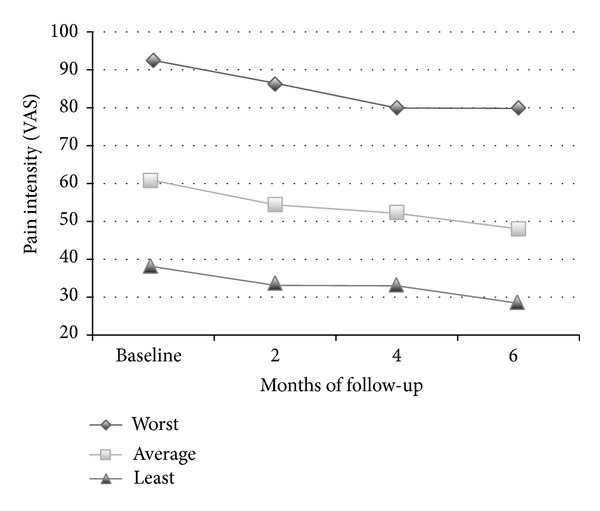
Pain intensity in the VAS scales before opioids and in subsequent 2-, 4-, and 6-month follow-ups.

All patients reported continuous pain; Figure 1 shows the mean values of worst, least, and average pain intensity in the four assessment phases. The most frequently administered analgesic was oral oxycodone (50.7%), followed by fentanyl (40%). No significant age and gender differences were found at baseline in worst, least, and average pain intensity.

In the repeated measures ANOVA, both worst and average pain as measured with VAS underwent a statistically significant (*P* < 0.001) reduction at 2-, 4-, and 6-month follow-ups; no statistically significant changes in VAS scores from baseline to 6-month follow-up were related to age, sex, and type of pain. The adherence to the treatment protocol was very high; only four out of 75 patients dropped the treatment due to side effects.

Approximately one-third of the patients (35%) reported the presence of at least one side effect at follow-up; the most frequently reported side effects were sleepiness (12%), nausea (11.3%%), and constipation (8.8%).

At 6-month follow-up, 46.2% of patients did not display any aberrant drug-related behaviors, 15.4% manifested only one, and the remaining 38.4% manifested two or more. In particular, 28.2% showed little interest in managing themselves and in rehabilitation, 24% frequently cancelled appointments, and 12.6% reported minimal or inadequate pain relief.

The urine toxicology screens with immunoassay did not identify the prescribed opioid in only one patient at the two-month follow-up, whereas it was identified in all patients at the 4- and 6-month follow-ups. In only one patient the result was questionable for illegal substances (suspect positivity for cocaine): so gas chromatography determination was made, which resulted negative.

As regards the psychological variables investigated in the pretreatment assessment (mean scores in Table 1) we found that, based on the personality profile obtained with MMPI-2, 24.5% of women and 27.7% of men had clinically significant scores (*T* scores ≥ 65) in at least one of the clinical scales with psychopathological content (Paranoia, Schizophrenia, and Hypomania). As for the affective-emotional variables, the depression score at BDI-2 was clinically significant (scores higher than 95th percentile) in 52.2% of patients; at STAI-trait anxiety 22.5% of patients showed a clinically significant score of anxiety.

#### 3.1.1. Analysis of the PMQ

The sample (*N* = 75) yielded a mean PMQ score of 25.4 (±10.35), a median score of 24, and a mode of 20 (lowest score = 8; highest score = 62). There were no significant age and gender differences in the PMQ scores.

Analysis of internal consistency produced an alpha coefficient of 0.77, indicating an adequate internal coherence; the test-retest reliability at 2 months was very high (*r* = 0.86;  *P* < 0.001). As for the predictive validity of the tool, highly significant correlations were found between the total PMQ score and the number of aberrant drug-related behaviors at 2-month (*r* = 0.58;  *P* < 0.01), 4-month (*r* = 0.67;  *P* < 0.01), and 6-month follow-ups (*r* = 0.52;  *P* < 0.01). The mean time required by the patient to complete the questionnaire was 12′16′′ (SD = 5.23; range: 4–30).

According to the cut-offs established by Dowling et al. [15], 41 subjects (54.7%) were at low risk, 16 (21.3%) at moderate risk, and 18 (24%) at high risk of drug misuse. The distribution in the three risk levels between men and women was comparable.

**Figure 2:**
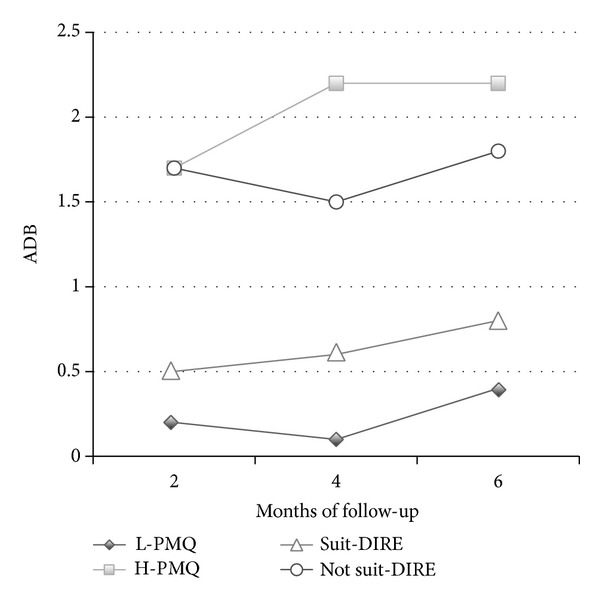
Mean frequency of aberrant drug-related behaviors (ADB) for high risk (H-PMQ) and low risk (L-PMQ) of drug misuse at PMQ and for low risk (Suit-DIRE) and high risk (Not suit-DIRE) at DIRE.

Nonparametric analysis of variance confirmed the presence of a significantly higher number of aberrant drug-related behaviors in the high-PMQ group compared to the low-PMQ group detected at 2-month (*Z* = 2.16;  *P* < 0.001), 4-month (*Z* = 2.64;  *P* < 0.001), and 6-month follow-ups (*Z* = 2.38;  *P* < 0.001) (Figure 2).

**Figure 3:**
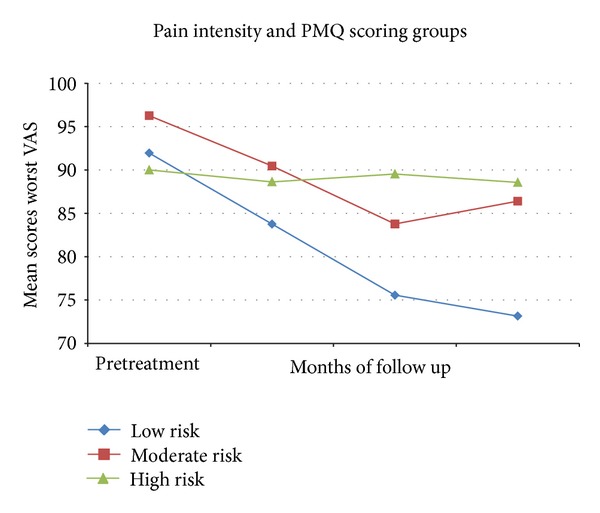
Mean scores of worst pain intensity (VAS) in the pretreatment phase and at 2-, 4-, and 6-month follow-ups relative to low (scores <25), moderate (<25–29>), and high (>30) PMQ scoring groups.

One-way repeated measures ANOVA showed a minor reduction in the worst pain intensity in the high-PMQ and moderate-PMQ groups when compared to the low-PMQ group (*F*_1,70_ = 6.75;  *P* < 0.01) (Figure 3).

The group was subsequently divided, according to the scores obtained at PMQ, in order to analyze the presence of any significant differences in the psychological variables considered in the study.

In general, Kruskal-Wallis analyses revealed significant differences (*P* < 0.01) between high- and low-PMQ scores in the following clinical scales: STAI-trait anxiety, BDI-total score, PRSS catastrophizing, MMPI hypochondriasis, MMPI depression, MMPI psychopathic deviate, MMPI Paranoia, MMPI psychasthenia, and MMPI Schizophrenia. As it can be seen in Table 1, the high-PMQ group showed significantly higher mean scores than the low-PMQ group in all these clinical scales. Moreover, 26% of patients with high PMQ and 12.3% of those with low PMQ obtained clinically significant scores (*T* scores ≥ 65) in at least one of the MMPI-2 clinical scales with psychopathological content (Paranoia, Schizophrenia, and Hypomania).

#### 3.1.2. Analysis of the DIRE

In the DIRE, the scores varied from 12 to 19, with a mean of 15.7 (SD = 1.8) and both median and mode of 16; there were no significant differences between the mean scores of men (14.8; SD = 1.5) and women (15.9; SD = 1.7). 85.3% of patients were found to be good candidates to start chronic opioid treatment according to the cut-offs established by Belgrade et al. [13]. No significant age and gender differences were found in the distribution of the two risk levels. The internal consistency of the Italian translation of the tool was 0.39, which is very low; the item that contributed least to the internal consistency of the DIRE was diagnosis (*α* if item deleted = 0.48).

In Figure 2 the mean frequencies of aberrant drug-related behaviors at follow-up for “suitable” and “not suitable” DIRE scores are shown, with significantly higher numbers of those behaviors in the “not suitable” group (*P* < 0.01) in all the assessment times. With regard to the predictive validity of the tool, significant correlations were found between a lower total DIRE score (higher risk of opioid misuse) and a higher number of aberrant drug-related behaviors detected at 2-month (*r* = −0.37;  *P* < 0.01), 4-month (*r* = −0.35;  *P* < 0.01), and 6-month follow-ups (*r* = −0.34; *P* < 0.01).

**Figure 4:**
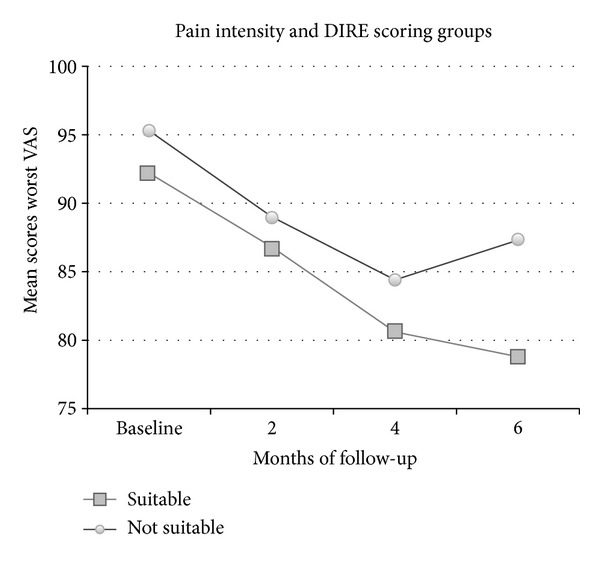
Mean scores of worst pain intensity (VAS) in the pretreatment phase and at 2-, 4-, and 6-month follow-ups relative to suitable (total score ≥14) and not suitable (total score ≤13) DIRE scoring groups.

Besides, “suitable” for opioid therapy patients showed a greater reduction in the worst pain intensity (VAS = 78.8; SD = 16.3) than “not suitable” patients (VAS = 87.3; SD = 11.3) (*F*_1,70_ = 5;  *P* < 0.05) at 6-month follow-up (Figure 4).

In order to investigate the presence of any significant differences in psychological variables, “suitable for opioid therapy” patients, based on DIRE score, were compared to “not suitable” for therapy patients.

Kolmogorov-Smirnov analysis underlined the presence of significantly higher mean scores (*P* < 0.01) in the “not suitable” group on two MMPI-2 scales: frequency (*Z* = 1.81) and Schizophrenia (*Z* = 1.66); a tendency toward significant differences (*P* < 0.05) was even found on STAI-trait anxiety (*Z* = 1.37) and BDI-total score (*Z* = 1.39) scales, with lower scores in the “suitable” for opioid therapy group (Table 1).

The mean time required by the multidisciplinary team to complete the tool was 7′37′′ (SD = 3.21; range: 1–16).

#### 3.1.3. PMQ, DIRE, and the Medical Risk Evaluation

As for the concurrent validity of the PMQ and DIRE, a significant negative correlation (*r* = −0.40;  *P* < 0.01) between high total PMQ scores (high risk of opioid misuse) and low DIRE total scores (not suitable) was found. As regards the 4 subscales of the medical risk evaluation, positive correlations (*P* < 0.01) were only found between the physician's estimate of the “opioid abuse” subscale and aberrant drug-related behaviors at 2-month (*r* = 0.32) and 6-month follow-ups (*r* = 0.39). No significant correlations emerged between the four scales of the medical risk evaluation and the PMQ total score. Instead, significant positive correlations (*P* < 0.01) were found between DIRE total score and the physician's evaluation of “compliance to treatment” (*r* = 0.33) and “treatment efficacy” (*r* = 0.34) while the DIRE total score correlates negatively with the physician's estimate of “opioid abuse” (*r* = −0.35) and risk of “underuse” (*r* = −0.34).

### 3.2. Discussion

The main aim of this study was to evaluate the preliminary validity of the Italian translation of the Pain Medication Questionnaire (PMQ) and the Diagnosis Intractability Risk and Efficacy Score (DIRE) in predicting the risk of opioid misuse in patients with chronic noncancer pain.

The total group of 75 patients had a mean age of 51.5 years, with a prevalence of females (76%), and a long history (roughly 10.5 years) of severe chronic pain condition, most often classified as nociceptive.

As for the psychological variables considered in the study, half of the samples reported clinically significant levels of depression and 22.5% of the patients displayed high levels of anxiety. In addition, there was a high incidence of elevation in patients' personality profiles: 24.5% of women and 27.7% of men reported clinically significantly high scores in at least one of the clinical content scales of the MMPI-2. These data are in line with the findings of many authors [24–26] and suggest the need to involve a psychotherapist in the assessment and treatment process of this population [9, 27].

According to the PMQ scores, about half of the group (53.3%) showed no particular risks of opioid misuse, while 24% of the subjects were found to be at high risk. A strong correlation was found between high-PMQ scores and the number of aberrant drug behaviors after 2, 4, and 6 months of treatment. No significant differences in demographic variables emerged between high- and low-PMQ scoring groups.

A further interesting result is represented by the lower pain reduction that occurred in patients at high risk of misuse, possibly due to inadequate use of the prescribed opioids. Patients classified as high risk of misuse, based on the cut-offs suggested by Dowling et al. [15], were found to be significantly more anxious and depressed and had a greater tendency to focus on physical symptoms and worry about them.

High-risk patients also reported higher mean scores and a higher percentage of clinically significant scores on the “Paranoia,” “Schizophrenia,” and “Hypomania” scales of the MMPI-2. Thus, the elevation of these scales, characterized by psychopathological traits, as well as a passive attitude in managing pain condition, and a greater tendency to produce pessimistic and catastrophic thoughts about pain are associated with a higher risk of opioid misuse. The association of high-PMQ scores, symptoms of depression, and psychological distress appears to be in line with previous findings [12, 28]. Furthermore, the PMQ has demonstrated adequate internal consistency and good reliability over the time. In addition, the short time required to complete the PMQ makes it easy to integrate it into clinical routine.

All these data suggest that the tool provides a reliable estimate of the risk of medication misuse and appears to be a reliable indicator that the clinician can use to plan regular treatment monitoring. The strong association of high-PMQ scores and the presence of symptoms of depression, anxiety, hypochondria, and catastrophizing suggest that opioid therapy needs to be combined with psychological treatments to reduce the affective and emotional distress and modify the patient's dysfunctional beliefs and behaviors related to the use of these drugs.

Also for the Italian translation of the DIRE, the results show that the total score is a good predictor of the risk of drug misuse: patients that obtained low scores in this instrument showed a significant greater frequency of aberrant drug-related behaviors in all the assessment times than those classified as good candidates for opioid therapy. Besides, suitable patients obtained a greater improvement in pain control than poor candidates at 6-month follow-up.

However, the DIRE has low internal consistency: this means that the items are very heterogeneous and that the tool probably has a multifactorial structure. Thus, our finding does not agree with the results reported by Belgrade et al. [13] in the original validation study, in which the DIRE displayed an internal consistency of 0.80.

Poor candidates for opioid treatment according to the DIRE score reported higher mean scores in anxiety, depression, and Schizophrenia-MMPI scales when compared to the good candidates. These results are consistent with previous findings, which identified the presence of psychiatric disorders as one of the most predictive factors of opioid abuse [29, 30]. Completing the DIRE requires a few minutes, but it must be preceded by a multidimensional and interdisciplinary assessment of the patient.

The two tools selected appear to be correlated; then both assess the same construct.

The physician's risk evaluation was found to be valid in estimating only the risk of opioid abuse at 2- and 6-month follow-ups; there were no significant relations between aberrant drug behaviors and medical risk evaluation of compliance with treatment, underuse of the drugs, or efficacy of treatment. Moreover, the correlations found between the physician's subjective evaluation for the risk of opioid abuse and the number of aberrant drug behaviors were weaker when compared to those obtained by using the PMQ and the DIRE. This result highlights the need to integrate clinical evaluation with the use of tools specifically created to assess the risk of opioid misuse in the chronic pain patient.

The urine toxicology screens were not able to identify the prescribed opioids only in one specimen out of 71. On the other hand, no illegal drugs were identified in any specimen. These data seem rather surprising compared with those reported by other authors in USA studies, who found up to 46.5% of the sample to have abnormal urine toxicology screen results [31–34].

The preliminary results seem to suggest that in our context the most frequent problem may be the underuse of opioid analgesics rather than their compulsive use and abuse as reported in the American literature: this interpretation is supported by the difficulty, frequently expressed by our patients and their families, in accepting these drugs for fear of addiction, loss of mental lucidity, or social stigmatization. This attitude seems to reflect the history of limited opioid use in Italy, which is one of the countries with the lowest morphine consumption in Europe [1, 7].

A further important objective of the multidisciplinary team is thus to assess patients' beliefs about these drugs so as to be able to modify any dysfunctional and unrealistic expectations. At the same time, the use of tools that allow identifying patients at risk of opioid misuse can reassure physicians that often tend to overestimate the risk of addiction. The short time required by the patient and the multidisciplinary team to complete their respective questionnaires supports the routinely use of these instruments in the clinical setting.

One may object that if universal precautions are used, stratification seems irrelevant. In fact this study reflects the Italian situation, where the Government has recently issued regulations aiming at promoting the use of analgesic opioids for therapeutic purposes. Consequently, we can expect the number of patients treated with opioids to grow significantly in the next years. The majority of these patients will be cared for by the General Practitioners who may not have the experience in the field or the time necessary to follow all of them with the maximal diligence according to the universal precautions. Hence it is important to carry out stratification of the risk of drug misuse before starting chronic opioid treatment, so that high-risk patients can be referred to interdisciplinary pain relief centers where they can be carefully monitored and trained on self-reliance.

In contrast with what is reported in the literature [31, 34], the adherence to the treatment protocol was very high; a possible explanation is that patients in our cohort had a long history of pain and they were probably highly motivated in the treatment prescribed by a specialized pain relief center. Besides, the frequent and scrupulous follow-up sessions that allowed rapid treatment and prevention of possible adverse events could have encouraged our subjects to maintain the therapy.

## 4. Conclusions

Our results support the adequate psychometric properties and clinical utility of the two screening measures employed. Besides, both PMQ and DIRE are more reliable than the clinician's evaluation alone in predicting the risk of opioid misuse, and the brevity in the administration allows their routinely use in a clinical setting. Both tests appear to be predictive of patient compliance and efficacy of analgesia in long-term opioid analgesic treatment. Nevertheless, the PMQ brevity of administration made it more useful for screening purposes in primary care setting, where the assessment is made in a short time and by General Practitioners. On the other hand, the DIRE required a multidisciplinary assessment, including an in-depth examination of psychosocial characteristics and attitude towards drugs and therapy. For this reason it could be more easily conducted in an interdisciplinary pain relief center to identify the specific support needs of the patient.

The limitations of this study are primarily the small number of subjects enrolled and the differing distribution of men and women. Despite these limitations, the study has allowed us to conduct preliminary analyses on the predictive validity and psychometric properties of the Italian translation of the PMQ and the DIRE, as well as to analyze affective-emotional variables and personality characteristics in patients treated with opioids.

Overall, our results highlight the need for a multidisciplinary assessment of patient candidates for chronic opioid treatment and for the use of tools specifically designed to predict the risk of misuse. Regular monitoring, psychological interventions, and urine drug screening will contribute to improve the compliance and outcome of long-term opioid use.

## Figures and Tables

**Table 1:** Patients characteristics: demographic, clinical, and psychological variables in the total group and for high and low risk in PMQ and DIRE.

Variables	Total sample *N* = 75	PMQ scoring group		*P* value^1^	DIRE scoring group		*P* value^2^
		L-PMQ^a^	H-PMQ^b^		Suit-DIRE^c^	NotSuit-DIRE^d^	
Age (years)							
Mean (SD)	51.5 (11.7)	52.7 (10.8)	53.6 (12.5)	—	52.1 (11.3)	50.3 (12.1)	—
Gender *N* (%)							
Female	57 (76)	34 (82.9)	13 (72.2)	—	50 (78.1)	7 (63.6)	—
Male	18 (24)	7 (17.1)	5 (27.8)	—	14 (21.9)	4 (27.3)	—
Marital status *N* (%)							
Married	56 (74.6)	33 (80.5)	15 (83.3)	—	47 (73.4)	9 (81.8)	—
Single	11 (15.3)	5 (12.2)	2 (11.1)	—	9 (14.1)	2 (18.2)	—
Separated/divorced	7 (9.3)	3 (7.3)	1 (5.6)	—	7 (10.9)	0 (0)	—
Widowed	1 (1.3)	0 (0.0)	0 (0.0)	—	1 (1.6)	0 (0)	—
Education (years) *N* (%)							
5	9 (12)	6 (14.6)	3 (16.7)	—	8 (12.5)	1 (9.1)	—
8	26 (34.6)	14 (34.2)	7 (38.9)	—	22 (34.4)	4 (36.4)	—
8–13	36 (48)	19 (46.3)	7 (38.9)	—	31 (48.4)	5 (45.4)	—
>13	4 (5.4)	2 (4.9)	1 (5.6)	—	3 (4.7)	1 (9.1)	—
Employment status *N* (%)							
Employed	28 (37.3)	16 (39.0)	6 (33.3)	—	24 (37.5)	4 (36.4)	—
Unemployed	2 (2.7)	0 (0.0)	2 (11.1)	—	2 (3.1)	0 (0)	—
Housewife	22 (29.3)	13 (31.7)	5 (27.8)	—	17 (26.6)	5 (45.4)	—
Retired	23 (30.7)	12 (29.3)	5 (27.8)	—	21 (32.8)	2 (18.2)	—
Type of pain *N* (%)							
Nociceptive	43 (57.3)	22 (53.6)	10 (55.6)	—	36 (56.2)	7 (631.6)	—
Neurophatic	9 (12)	7 (17.1)	2 (11.1)	—	9 (14.1)	1 (9.1)	—
Mixed	23 (30.7)	12 (29.3)	6 (33.3)	—	19 (29.7)	3 (27.3)	—
Duration of pain (months)							
Mean (SD)	125.9 (105.9)	138.9 (107.7)	106.3 (93.7)	—	128.4 (99.1)	118.7 (121.0)	—
Psychological variables							
STAI Y2							
Mean (SD)	50.6 (9.3)	46.7 (6.9)	56.5 (10.4)	<0.01	49.6 (8.4)	55.9 (11.9)	<0.05
BDI-2 total							
Mean (SD)	21 (10.7)	16.5 (8.6)	28.0 (11.5)	<0.01	19.2 (9.2)	30.5 (13.6)	<0.05
PRSS catastrophe							
Mean (SD)	3.0 (1.1)	2.6 (1.1)	3.8 (0.7)	<0.01	2.9 (1.1)	3.6 (0.9)	—
MMPI hypochondriasis∗							
Mean (SD)	17.2 (5.2)	15.3 (3.9)	20.2 (4.7)	<0.01	16.9 (5.5)	19.1 (4.4)	—
MMPI depression∗							
Mean (SD)	29.8 (5.4)	28.2 (4.7)	32.0 (6.1)	<0.01	29.2 (5.1)	32.7 (6.0)	—
MMPI hysteria∗							
Mean (SD)	31.6 (4.8)	30.4 (4.4)	32.9 (4.8)	—	19.6 (5.1)	32.7 (6.0)	—
MMPI psychopathic D∗							
Mean (SD)∗	20.3 (5.3)	18.7 (5.1)	23.3 (4.8)	<0.01	19.6 (5.1)	24.0 (4.6)	—
MMPI-mascul./feminin.∗							
Mean (SD)∗	31.4 (4.6)	30.3 (4.2)	31.7 (4.5)	—	30.5 (4.1)	31.6 (4.0)	—
MMPI Paranoia∗							
Mean (SD)	11.8 (3.4)	10.3 (2.2)	14.3 (2.7)	<0.01	11.2 (3.0)	14.8 (3.9)	—
MMPI psychasthenia∗							
Mean (SD)	20.7 (8.3)	18.1 (8.6)	23.8 (7.1)	<0.01	19.1 (7.8)	27.0 (7.8)	—
MMPI Schizophrenia∗							
Mean (SD)	20.8 (10.4)	17.3 (9.7)	25.8 (10.5)	<0.01	18.8 (9.7)	29.5 (9.6)	<0.01
MMPI Hypomania∗							
Mean (SD)	18.5 (4.3)	17.8 (4.4)	19.4 (4.2)	—	18.5 (4.3)	18.6 (3.2)	—
MMPI social introver.∗							
Mean (SD)	32.6 (7.2)	32.6 (6.3)	33.5 (8.7)	—	31.3 (6.3)	39.5 (7.6)	—

^a^L-PMQ group, *n* = 41 (PMQ total score <25); ^b^H-PMQ group, *n* = 18 (PMQ total score ≥30).

^c^Suitable-DIRE group, *n* = 64 (DIRE total score ≥14). ^d^Not suitable-DIRE group, *n* = 11 (DIRE total score ≤13).

∗Row total scores (no *K*-correction).

*P* value: significant differences in mean scores between PMQ scoring groups (*P* value^1^) and between DIRE scoring groups (*P* value^2^).
